# TRPV1 Agonist, Capsaicin, Induces Axon Outgrowth after Injury via Ca^2+^/PKA Signaling

**DOI:** 10.1523/ENEURO.0095-18.2018

**Published:** 2018-05-30

**Authors:** Erin Frey, Scott Karney-Grobe, Trevor Krolak, Jeff Milbrandt, Aaron DiAntonio

**Affiliations:** 1 Department of Developmental Biology, Hope Center for Neurological Disorders, Washington University School of Medicine, St. Louis, MO 63110, USA; 2 Department of Genetics, Hope Center for Neurological Disorders, Washington University School of Medicine, St. Louis, MO 63110, USA

**Keywords:** Axon Regeneration, Capsaicin, Neurite Outgrowth, PKA, Preconditioning, TRPV1

## Abstract

Preconditioning nerve injuries activate a pro-regenerative program that enhances axon regeneration for most classes of sensory neurons. However, nociceptive sensory neurons and central nervous system neurons regenerate poorly. In hopes of identifying novel mechanisms that promote regeneration, we screened for drugs that mimicked the preconditioning response and identified a nociceptive ligand that activates a preconditioning-like response to promote axon outgrowth. We show that activating the ion channel TRPV1 with capsaicin induces axon outgrowth of cultured dorsal root ganglion (DRG) sensory neurons, and that this effect is blocked in TRPV1 knockout neurons. Regeneration occurs only in NF200-negative nociceptive neurons, consistent with a cell-autonomous mechanism. Moreover, we identify a signaling pathway in which TRPV1 activation leads to calcium influx and protein kinase A (PKA) activation to induce a preconditioning-like response. Finally, capsaicin administration to the mouse sciatic nerve activates a similar preconditioning-like response and induces enhanced axonal outgrowth, indicating that this pathway can be induced *in vivo*. These findings highlight the use of local ligands to induce regeneration and suggest that it may be possible to target selective neuronal populations for repair, including cell types that often fail to regenerate.

## Significance Statement

After injury or neurodegenerative disease, axons need to regenerate to restore function. Unfortunately, no current therapies achieve this goal. We performed a drug screen in adult sensory neurons to identify agents that effectively reprogram neurons into a progrowth state. We demonstrate that capsaicin, a TRPV1 agonist, activates a pro-axon growth program. This mechanism requires calcium influx and PKA activity and leads to the activation of the pro-regenerative transcription factor CREB. Hence, activation of ion channels can promote axon regeneration, an exciting finding, since many ion channels are druggable targets. Moreover, these findings suggest an approach for enhancing axon regeneration in selective populations of neurons.

## Introduction

Axon regeneration is required to restore function after nervous system damage. CNS neurons are notoriously regeneration-resistant, and even some subtypes of peripheral nervous system (PNS) neurons have a decreased capacity to regenerate. Identifying strategies to improve regeneration of otherwise regeneration-resistant neurons could be beneficial after CNS or PNS damage.

After injury, local signaling and cytoskeletal rearrangement establishes new growth cones (Bradke et al., 2012). This step is necessary but not sufficient for long-distance regeneration. Most dorsal root ganglion (DRG) neurons initiate a robust pro-regenerative transcriptional program that reprograms neurons into a regenerative state (Tedeschi, 2011). Prior activation of this program by a conditioning lesion removes that delay and accelerates regeneration following a second test lesion (McQuarrie and Grafstein, 1973; McQuarrie et al., 1977; Smith and Skene, 1997). This preconditioning effect results from activation of the pro-regenerative program by the conditioning injury so that, at the time of the second injury, neurons are already in a pro-regenerative state. Mimicking the preconditioning response is an exciting therapeutic strategy to enhance regeneration, particularly in regeneration-resistant neurons. The cAMP stimulator forskolin was the first discovered agent with this capacity (Kilmer and Carlsen, 1984). Further studies determined that elevating cAMP/PKA signaling not only stimulated regeneration of sensory neurons on growth-permissive substrates, but also stimulated regeneration over inhibitory substrates as well as into the CNS *in vivo* (Cai et al., 1999; Qiu et al., 2002; Gao et al., 2004). Mechanistically, PKA phosphorylates CREB to induce a pro-regenerative transcriptional program (Cai et al., 2002; Gao et al., 2004). Drugs that manipulate these existing pro-regenerative pathways offer an exciting strategy for nervous system repair.

We set out to perform a screen to identify drugs that mimic preconditioning in cultured adult sensory neurons. Importantly, we sought drugs that work in the absence of injury. Traditionally, regeneration screens apply drugs after injury and assess growth over inhibitory substrates. This strategy has successfully identified signaling cascades such as the PKC and AKT pathways, as well as drugs that alter microtubule density and dynamics (Gao et al., 2010; Samara et al., 2010; Usher et al., 2010). However, these screens were not ideal for finding drugs that mimic the preconditioning phenomenon in the absence of injury. First, the drugs were present as the axons were growing, so it was difficult to know whether these drugs (a) act locally at the growth cone to promote outgrowth or (b) mimic preconditioning to activate the pro-regenerative program. Second, because dissection of DRG neurons from the animal is an injury, the drugs were competing with the endogenous injury-signaling program. Hence, the drugs would need to enhance regeneration beyond the natural injury stimuli to be identified. As an alternative approach, we sought to design a screen in which drugs would be applied in the absence of injury signaling and before the period of axonal outgrowth. To achieve this, we used our previously described assay in which compounds can be applied to neurons in the absence of injury and washed out, and neurons can be replated to initiate new neurite outgrowth (Frey et al., 2015). This allows us to distinguish between the activation phase and growth phase of axonal outgrowth and so identify drugs that mimic preconditioning and “reprogram” uninjured DRG neurons into a regenerative state.

After screening 480 test compounds, we discovered that TRPV1 agonists mimicked preconditioning. Capsaicin upregulated the pro-growth transcription factor phosphorylated CREB and induced PKA-dependent axon outgrowth. Local application of capsaicin to the sciatic nerve also induced axon outgrowth, indicating that TRPV1 activation within the axon can induce this signaling pathway. This demonstrates that ion channel ligands can target selective populations of neurons for regeneration. Furthermore, these results illustrate the utility of this assay in identifying novel pro-regeneration drugs that function *in vivo*.

## Materials and Methods

### Animals

Adult CD1 mice were purchased from Charles River. TRPV1 knockout (KO) mice (Caterina et al., 2000) were provided by Dr. Robert Gereau of Washington University in St. Louis. Both male and female mice were used. Mouse husbandry was performed under the supervision of the Division of Comparative Medicine at Washington University.

### Primary DRG neuron culture

DRG neurons were cultured as described (Frey et al., 2015). Briefly, all DRG were dissected from adult mice and incubated in 0.35 mg/ml Liberase Blendzyme (Roche), 10 mg/ml bovine serum albumin (Sigma), and 0.6 mg/ml DNase (Sigma) for 15 min, followed by 0.05% trypsin-EDTA (Invitrogen) for 15 min at 37°C. Neurons were dissociated in DMEM (Invitrogen) media containing 10% FBS (Invitrogen), 100 U/ml penicillin, and 100 µg/ml streptomycin (Invitrogen) and plated on culture dishes or glass chamber slides coated with 10 mg/ml of each PDL and laminin (Sigma). Neurons were maintained at 37°C with 5% CO_2_. Media was changed on day *in vitro* 1 (DIV1), and 4 and 10 µm AraC (Sigma) was added.

### Drug treatment for axon regeneration screen

ICCB Known Bioactives Library (Maxene Iligan) was provided from the high-throughput screening core at Washington University in St. Louis. This library contains 480 drugs dissolved in DMSO. Drugs were diluted in media and applied at 1:5000 and 1:1000 dilutions. This gave final concentrations for most drugs between 1 and 30 µm. DMSO and 1 µm Nocodazole (Sigma) were used as negative and positive controls, respectively (Valakh et al., 2015). Ten negative and positive controls were included on each 96-well plate. Nocodazole-treated wells were normalized to DMSO-treated wells from the same plate to control for plate-to-plate variability. If nocodazole failed to induce robust neurite outgrowth, all data from that plate were excluded, and those compounds were tested on new plates from a fresh culture. Over the entire screen, DMSO-treated neurons had a mean neurite length of 281 ± 29 µm/neuron compared to nocodazole-treated neurons, which extended neurites to 1000 ± 85 µm/neuron.

### Capsaicin treatment

Drugs were applied as described in the figures. Capsaicin (10 µm, ApexBio or Cayman Chemical) was applied for 10 min or 24 h. Inhibitors (capsazepine, Cayman Chemical; EGTA, Sigma; H89, Sigma) were pretreated for 10–30 min before capsaicin treatment. All inhibitors were present during capsaicin treatment. H89 was also present during the rest of the 24-h activation phase. All drugs were removed immediately before replating. For phospho-CREB immunostaining, capsaicin was removed 20 min before fixation. For *in vivo* experiments, surgifoam was dissolved in 200 µm capsaicin or DMSO and applied to the sciatic nerve.

### Sciatic nerve surgeries

Mice were anesthetized with isofluorane. The surgical site was shaved and cleaned with betadine solution. A small incision was made along one thigh, and the sciatic nerve was exposed. Surgifoam was applied or the nerve was crushed. For nerve crush, number 5 forceps were used to crush the nerve for 40 s (two 20-s orthogonal crushes). After 2 d, all mice were sacrificed, and L4–6 DRG were collected for culture.

### Replating assay

After drug treatment, neurons were washed with DMEM, then treated with 0.025% Trypsin-EDTA for 5 min at 37°C. Neurons were then dissociated and pipetted up and down to strip off axons before being replated onto PDL/laminin-coated 96-well plates (for screen) or glass chamber slides (for manual assay) in culture media.

### Immunocytochemistry and dye staining

After treatment, cells were fixed with 4% paraformaldehyde (Electron Microscopy Services) in PBS containing 0.1% Triton (PBST) for 20 min at room temperature. Neurons were blocked in PBST containing 10% normal goat serum. Primary antibody was incubated overnight at 4°C, washed 3 times with PBST, incubated in secondary antibody for at least 1 h at room temperature, and washed again with PBST before being coverslipped with Vectashield (Vector Laboratories). Commercial antibodies used were as follows: mouse anti-Tuj1 (Covance, 1:500; RRID: AB_2313773), rabbit anti-pCREB (Cell Signaling Technologies, 1:500, RRID: AB_2561044), rabbit anti-NF200 (Sigma, 1:500, RRID: AB_477272), goat anti-mouse AF488 (Life Technologies 1:1000, RRID: AB_138404), and goat anti-rabbit Cy3 (Jackson Immuno, 1:1000, RRID: AB_10563288). SCG10 antibody was purified from anti-SCG10 rabbit antiserum (Novus Biologicals) using the SulfoLink Kit (Thermo Fisher). Ethidium homodimer (2 µM, Sigma) and TMRM (50 nM, Sigma) were incubated for at least 15 min before imaging.

### Imaging and data analysis

For SCG10 and phospho-CREB, cells were imaged on a Leica DMI 4000B Confocal microscope using 20× or 40× oil objectives. Images are maximum projections of confocal stacks. Identical confocal settings were used for samples within an experiment. Mean intensity of SCG10 in the soma or phospho-CREB in the nucleus was quantified using ImageJ to determine the mean gray value and normalized to the negative control (DMSO) within each experiment. 50–100 neurons were imaged for each group per experiment. For manual axon outgrowth, at least 100 neurons were imaged per group per experiment using a Leica DFC7000T color fluorescence camera. Neurite length was quantified by tracing the longest neurite from each cell using the NeuronJ plugin for ImageJ (Meijering et al., 2004; RRID: SCR_002074). For NF200 experiments, NF200 immunostaining was imaged in addition to βIII-tubulin. NF200 intensity and neurite length were analyzed for at least 300 neurons per experiment and classified as NF200 positive or negative. Cells with intermediate NF200 intensity were excluded from analysis. For the axon regeneration screen, plates were imaged on the Operetta High-Content Imaging System (PerkinElmer) at 20× magnification. ∼50 images were taken from each well. Images were processed in Cell Profiler (Carpenter et al., 2006; RRID: SCR_007358). We generated a pipeline to count cells using Hoechst staining. Neurons were identified as nuclei that were βIII-tubulin positive. This yielded total cell and neuronal cell counts. The area of the somas was subtracted from the βIII-tubulin channel so that only neurites would be quantified. Cell Profiler then quantified the total neurite length in each image. Mean neurite length was calculated from the total neurite length from all neurons in each well then divided by the total number of neurons from the same well. This was then normalized to the neurite outgrowth from negative controls. Cell counts were used to rule out drugs that induced toxicity in the screen. Ethidium homodimer and TMRM were also imaged on the Operetta High-Content Imaging System. At least 100 neurons were imaged from each condition to assess cell health. TMRM intensity was quantified similarly to phospho-CREB and SCG10 immunostaining. For viability, DRG neurons were identified and counted in bright-field images. Cells that were positive for ethidium homodimer were labeled “dead.” For *in vivo* axon regeneration, nerves were stained for βIII-tubulin and SCG10 and imaged on the Leica DMI 4000B Confocal using the 20× oil objective. The regeneration index was measured as the distance where SCG10 intensity was 50% of the level at the injury site, as we have previously described (Shin et al., 2012a, 2014).

### Experimental design and statistical analysis

**Figure 1.**
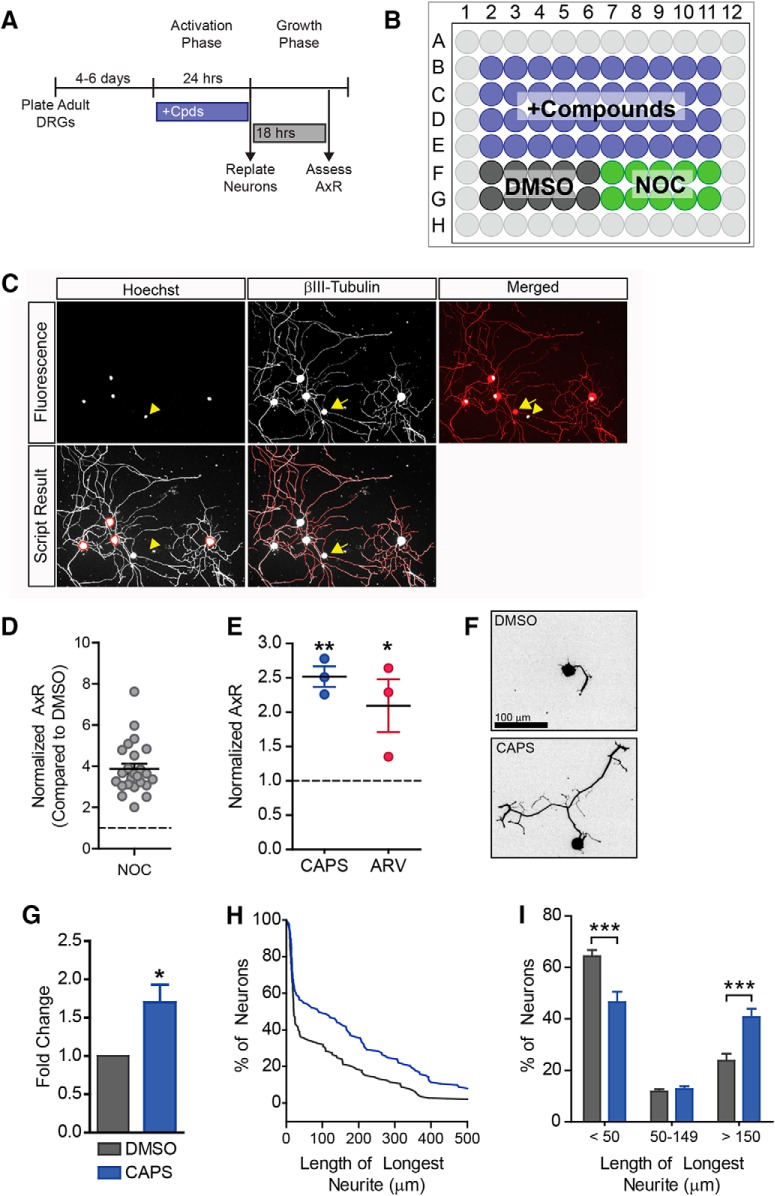
Drug screen identifies TRPV1 agonists as progrowth drugs. A drug screen was performed to identify drugs that could induce a regenerative state. ***A***, ***B***, Experimental design. After 4–6 d in culture, neurons were treated for 24 h, then drugs were washed out and neurons were replated. After 18 h of growth, neurons were fixed and stained to assess axon growth. 40 compounds were tested per plate with 10 negative controls (DMSO) and 10 positive controls (NOC). Outer wells were filled with water to minimize evaporation. ***C***, Fluorescence images for Hoechst (nuclei) and βIII-tubulin (neuron specific marker) are shown. Note that the non-neuronal cell (βIII-tubulin negative nucleus, yellow arrowhead) is not counted. Note the debris (yellow arrow) is not traced. Neurite tracings are shown in red. ***D***, Nocodazole induces robust growth in this high-throughput assay. Nocadazole-treated wells were normalized to DMSO-treated wells from the same plate, therefore DMSO = 1 (dashed line). Each data point represents one plate. Twenty-four plates were used in the screen. ***E***, In this screen, two TRPV1 agonists: capsaicin (CAPS) and arvanil (ARV) increased axon outgrowth compared to DMSO, (*n* = 3 independent experiments). Capsaicin was validated by analyzing axon growth from individual neurons (10 µM, *n* = 5 independent experiments). Representative images are shown (***F***). Capsaicin induced a ∼1.7-fold increase in mean neurite length (***G***) and shifted the distribution of cells toward having longer neurites (***H***, ***I***). A representative cumulative distribution is shown in ***H***.

**Figure 2.**
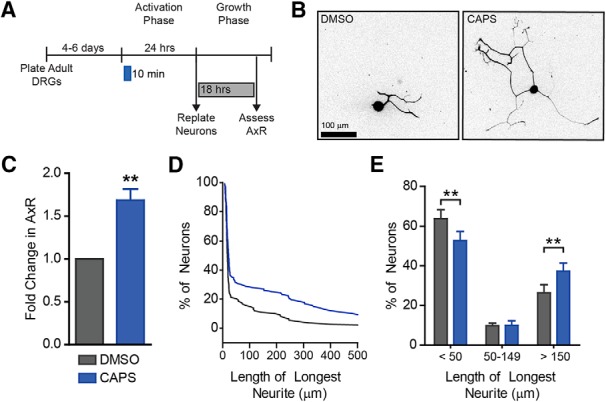
Capsaicin pulse induces axon outgrowth. Since TRPV1 opens transiently in response to stimulation, neurons were treated transiently with a 10-min pulse of capsaicin (***A***). After 10 min, capsaicin was removed. Neurons were replated 24 h later and grown for 18 h. Representative images are shown (***B***). Capsaicin induced a ∼1.7-fold increase in mean neurite length (***C***) and a subset of neurons extended longer neurites (***D***, ***E***). A representative cumulative distribution is shown in ***D***. *n* = 5 independent experiments.

**Figure 3.**
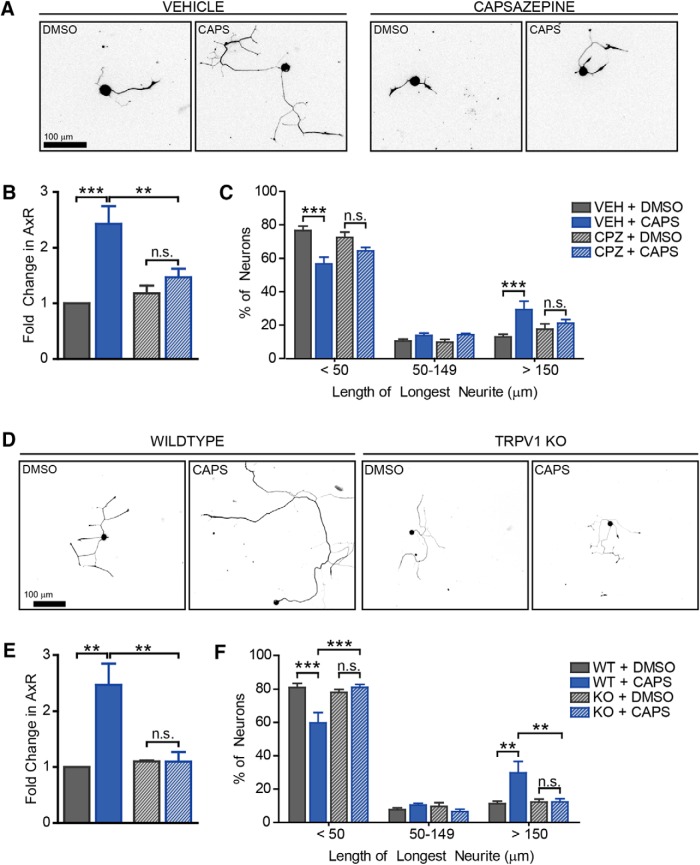
TRPV1 is required for capsaicin-induced outgrowth. We used capsazepine, a capsaicin antagonist, and TRPV1 KO mice to determine if capsaicin-induced outgrowth required TRPV1. Neurons were treated with capsazepine (CPZ, 10 µM) for 10 min before capsaicin. After 10 min of capsaicin treatment, both drugs were washed away. Neurons were replated after 24 h and grown for 18 h. Representative images are shown (***A***). Capsazepine blocked capsaicin-induced outgrowth (***B***) and the increased proportion of neurons with longer neurites (***C***). *n* = 4 independent experiments. WT and TRPV1 KO neurons were treated as in Fig. 2*A*. TRPV1 KO completely blocked capsaicin-induced outgrowth in both measures, mean neurite length (***D***, ***E***) and distribution (***F***). Data are from 4 independent experiments.

**Figure 4.**
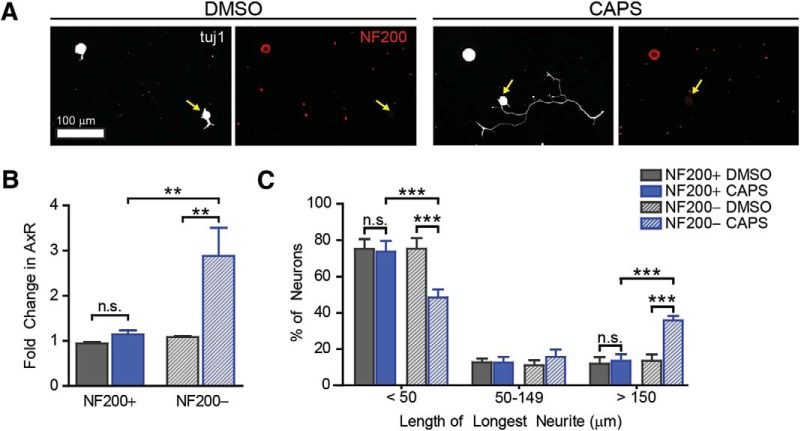
Capsaicin induces axon outgrowth in NF200^–^ nociceptive neurons. To determine if outgrowth was being induced in non-nociceptive (NF200^+^) or nociceptive (NF200^–^) neurons, we performed the same experiment described in Fig. 2*A*, then colabeled cells for βIII-tubulin and NF200. Yellow arrows indicate NF200^–^ neurons (***A***). NF200^–^ neurons (yellow arrow) showed increased mean neurite length (***B***) with a significant shift in the percentage of cells extending long neurites (***C***). No change was observed in NF200^+^ neurons treated with capsaicin. Mean neurite length was normalized to the entire population (NF200^+^ and NF200^–^) of DMSO-treated neurons. *n* = 4 independent experiments.

**Figure 5.**
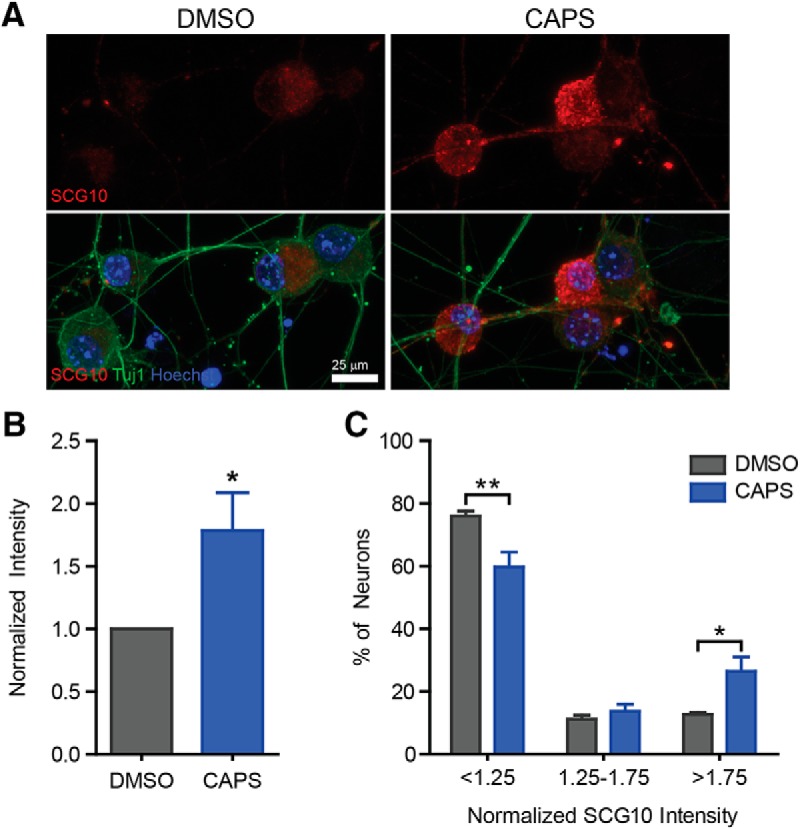
Capsaicin upregulates the regeneration marker SCG10. To determine if the pro-regeneration program had been activated, we assessed upregulation of the regeneration marker SCG10. Neurons were treated with capsaicin for 10 min. 24 h later, neurons were fixed and stained for SCG10. Representative images (***A***) show an increase in mean SCG10 intensity (***B***) as well as an increase in the percentage of neurons that have robust levels (>1.75) of SCG10 in the cell body (***C***). *n* = 5 independent experiments.

**Figure 6.**
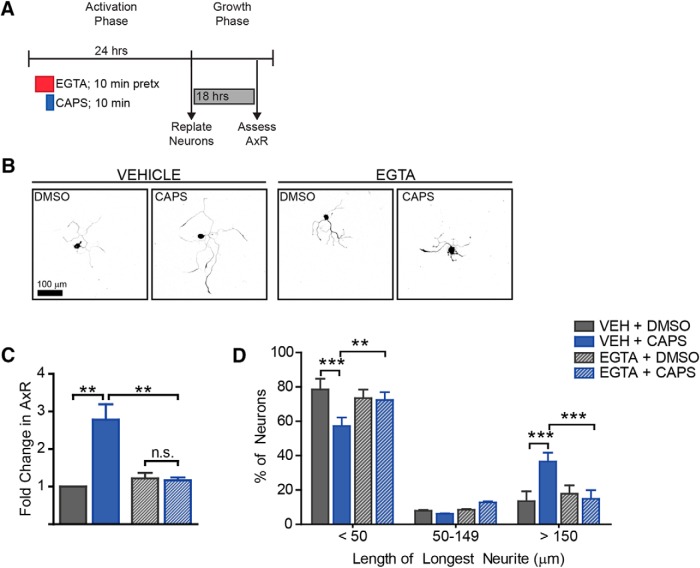
Extracellular calcium is required for capsaicin-induced outgrowth. To determine if Ca^2+^ influx was required for outgrowth induced by capsaicin, neurons were treated with EGTA to chelate extracellular calcium for 10 min before capsaicin addition. After the 10-min capsaicin pulse, both drugs were removed, and neurons were cultured for 24 h before replating (***A***). Representative images (***B***) illustrate that EGTA robustly blocked capsaicin-induced outgrowth. EGTA blocked the increase in mean neurite length (***C***) and the increased number of neurons with longer neurites (***D***). *n* = 3 independent experiments.

**Figure 7.**
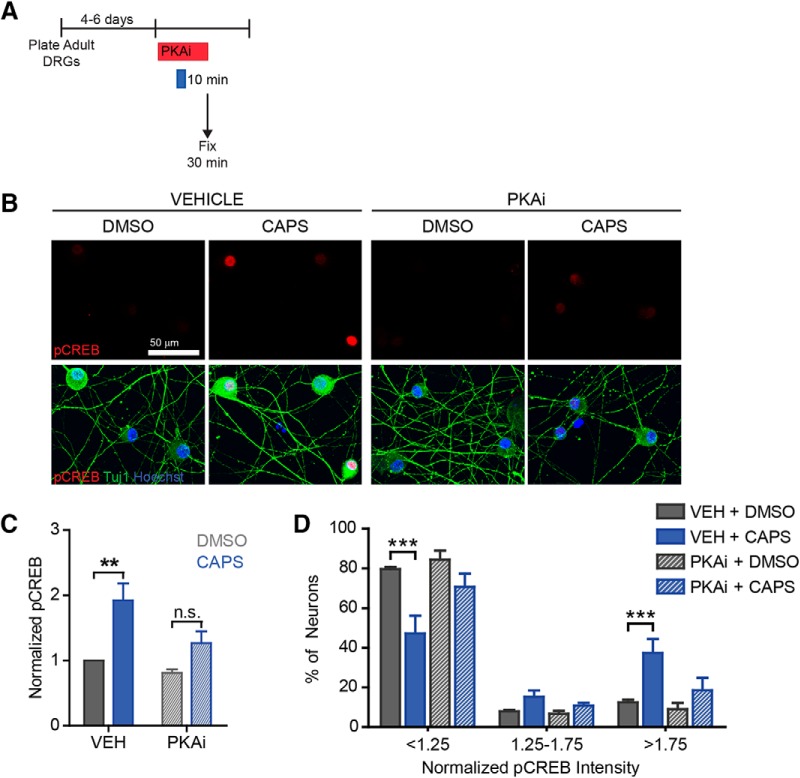
Capsaicin induces PKA-dependent pCREB. To identify the signaling pathway involved in capsaicin-induced outgrowth, we assessed phosphorylation of the pro-regenerative transcription factor CREB. ***A***, Vehicle or H-89 (PKAi, 5 µM) was applied 30 min before DMSO/capsaicin treatment. After 10 min, drugs were washed out, and vehicle/PKAi were reapplied. 20 min later neurons were fixed and stained (***B–D***). Overall, there was an ∼2-fold increase in pCREB intensity (***C***, *n* = 4 independent experiments). ***D***, The distribution showed that only a subset of neurons had robust pCREB upregulation. Capsaicin-induced pCREB was decreased by PKA inhibition.

**Figure 8.**
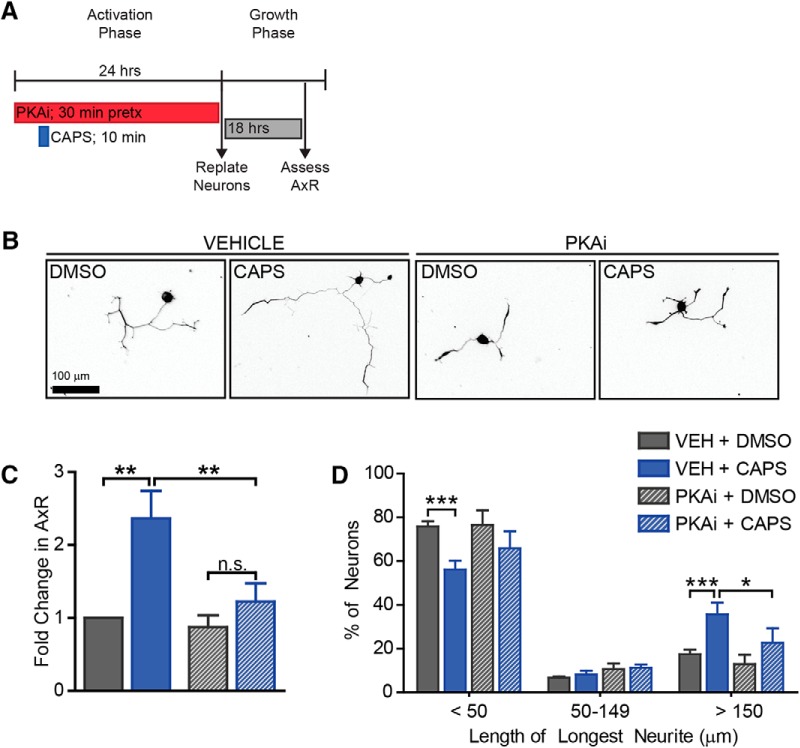
Capsaicin-induced axon growth is PKA-dependent. To determine if PKA was required for axon growth, we pretreated neurons with PKAi for 30 min before capsaicin treatment. PKAi remained on the cells during capsaicin treatment and during the rest of the activation phase (***A***). PKAi diminished the increase observed in mean neurite length (***B***,***C***) and the increase in the proportion of neurons with long neurites (***D***). *n* = 3 independent experiments.

**Figure 9.**
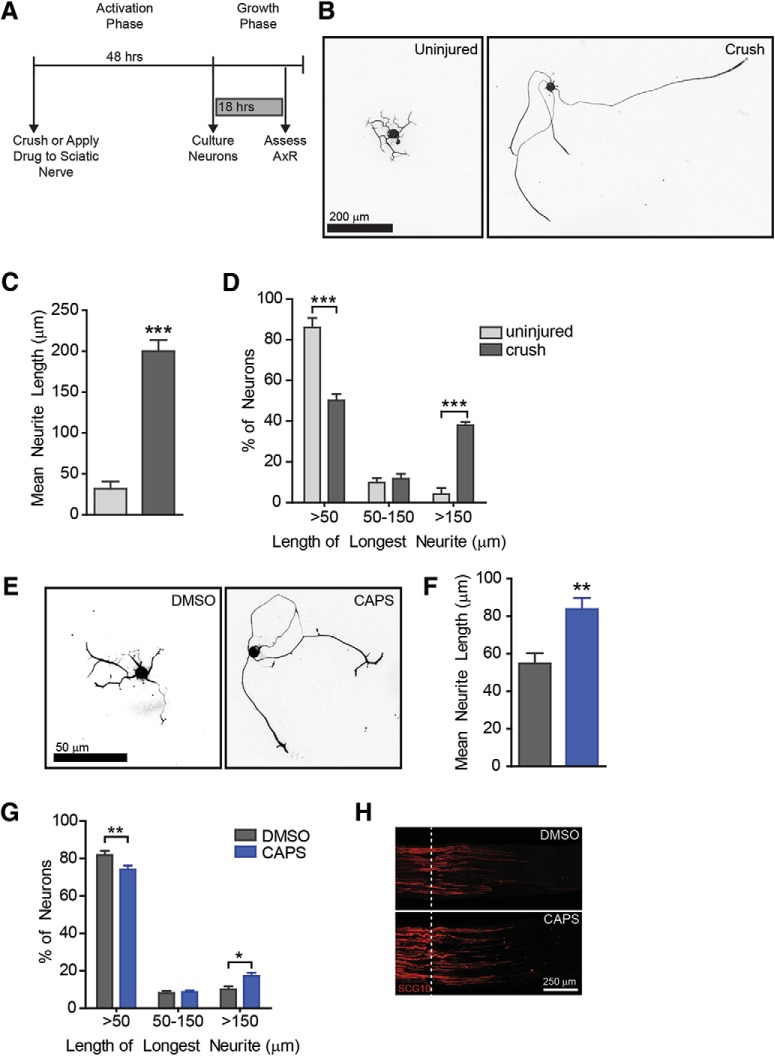
Capsaicin applied to the sciatic nerve induces axonal outgrowth. Progrowth signaling can be activated *in vivo* and observed *in vitro* (***A***). Preconditioning nerve crush induces robust axon outgrowth (***B–D***, *n* = 3). To determine if this capsaicin pathway is active *in vivo*, capsaicin was applied locally to the mouse sciatic nerve. After 2 d, L4/5 DRG were removed and cultured for 18 h to determine if local capsaicin had induced a pro-regenerative program. Representative images (***E***) show that capsaicin treatment induced axon outgrowth and increased mean neurite length (***G***) and shifted a subset of neurons toward longer growth (***G***). *n* = 7 independent experiments. ***H***, Representative images of sciatic nerve show that capsaicin was insufficient to induce regeneration in the sciatic nerve.

Previous studies have indicated that a sample size of at least 3 independent experiments is required to detect significant changes between control (DMSO) and experimental conditions, assuming α = 0.05. One independent experiment is defined as a single DRG culture. All conditions were tested in neurons from the same culture; therefore all experimental conditions were compared to their control counterparts. For *in vivo* experiments, 14 mice were used. Half were treated with DMSO and half were treated with capsaicin. All data are shown as mean ± SEM. Student’s *t* test, one-way ANOVA with Tukey *post hoc* test, and two-way ANOVA with Bonferroni *post hoc* test were used to determine if differences were statistically different. Student’s *t* test was performed on the following data: Figs. 1*G* (paired), 2*C* (paired), 4*B* (paired), 8*C* (unpaired). One-way ANOVA with Tukey *post hoc* test was performed on: Figs. 1*E*, 3*B*, 3*E*, 6*C*, 7*C*, 8*C*. Two-way ANOVA with Bonferroni *post hoc* test was used with Figs. 1*H*, 2*D*, 3*C*, 3*F*, 4*B*, 4*C*, 5*C*, 6*D*, 7*D*, 8*D*, 9*D* . In figures, *, **, and *** indicate *p*-values <0.05, <0.01, and <0.001, respectively. Exact *p*-values are given in the text.

## Results

### An automated cellular imaging assay identifies pro-regenerative drugs

We previously characterized an *in vitro* axon regeneration assay capable of identifying drugs that mimic preconditioning and activate the pro-regenerative program (Frey et al., 2015). Adult DRG neurons are cultured for 4–6 d to allow the axon regeneration program activated by the initial dissection to turn off (Frey et al., 2015). Drugs are applied for 24 h (activation phase), then are washed away, and neurons are replated and grown for 18 h (growth phase) in the absence of drug (Fig. 1*A*). This allows us to identify drugs that mimic preconditioning and reprogram neurons into a regenerative state rather than drugs that act locally to regulate growth cone and cytoskeletal dynamics. We used this assay to screen ∼480 drugs for their ability to induce axonal outgrowth.

Previously, we discovered that nocodazole, a drug that disrupts microtubules, activates the pro-regenerative kinase DLK and induces robust regeneration (Valakh et al., 2015). Thus, we used nocodazole as a positive control (Fig. 1*A–D*). Nocodazole controls were normalized to DMSO controls from the same plate to control for plate-to-plate variability. Nocodazole consistently induced robust growth on each plate, giving a mean value of 3.9-fold over DMSO for the entire screen (Fig. 1*D*, *n* = 24 plates, df = 46, *p* = 0.0001). We identified several drugs that would be expected to induce regeneration. These included cytoskeletal disrupting agents such as podofilox, mebendazole, febendazole, and nocodazole, as well as the PKA stimulator, forskolin (data not shown). We also identified capsaicin and arvanil, 2 TRPV1 agonists, that induced a 2.5- and 2.1-fold increase in neurite outgrowth, respectively (Fig. 1*E*, df = 6, capsaicin *p* = 0.01, arvanil *p* = 0.04). Because TRPV1 had not previously been linked to promoting axonal regeneration, we explored this finding further.

Our automated assay allowed us to screen hundreds of drugs, but it has limitations. For example, the pipeline is not able to determine which neurites belong to which neuron. Instead, the neurite length analysis normalizes total neurite length to the total number of neurons, leaving us blind to the behavior of individual neurons. This is of particular import, because TRPV1 is expressed in only a subset of nociceptive neurons. To get a clearer picture of how the population of neurons responded to these drugs, we measured mean neurite length of individual neurons. Capsaicin induced a ∼1.7-fold increase in mean neurite length (Fig. 1*F*,*G*, df = 4, *p* = 0.04). While not all neurons extended long neurites, capsaicin shifted the distribution of neurons with neurites (Fig. 1*H*): fewer cells had short neurites, <50 µm (df = 12, *p* = 0.001) and more neurons had longer neurites, >150 µm (df = 12, *p* = 0.001, Fig. 1*I*). We conclude that capsaicin induces axon outgrowth in a subset of neurons.

### Acute capsaicin induces axon outgrowth *in vitr*o

Capsaicin is an agonist for the nociceptive ion channel TRPV1 (Caterina et al., 1997). TRPV1 opens transiently in response to stimulation. Therefore, we tested whether transient capsaicin exposure was sufficient to promote outgrowth. Neurons treated with capsaicin for only the first 10 min of the activation phase showed a ∼1.7-fold increase in axon outgrowth compared to DMSO (Fig. 2*A–C*, df = 4, *p* = 0.007). This was similar to the increase we saw when capsaicin was applied for 24 h. The increased neurite length was apparent as a shift in the distribution of neurons with long neurites (Fig. 2*D*). Indeed, capsaicin induces a decrease in neurons with short neurites and an increase in neurons with long neurites (Fig. 2*E*, df = 12, *p* = 0.002, 1, 0.002 for each bin, respectively). These results are consistent with a role of an ion channel such as TRPV1 in stimulating a preconditioning-like response.

### TRPV1 is required for capsaicin-induced outgrowth

Capsaicin is a well-known agonist for TRPV1. Therefore, we hypothesized that TRPV1 mediates capsaicin-induced axon outgrowth. We first tested this hypothesis using the well-validated TRPV1 inhibitor capsazepine (CPZ). CPZ was added 10 min before capsaicin and left on through the 10-min capsaicin treatment. After capsaicin treatment, both drugs were washed out. Neurons were replated 24 h later and grown for an additional 18 h. CPZ blocked the 2.4-fold increase in outgrowth caused by capsaicin (Fig. 3*A*,*B*, df = 9, vehicle *p*-value = 0.0005, CPZ *p*-value = 0.6, vehicle vs. CPZ capsaicin *p*-value = 0.008). Capsazepine also blocked the shift in distribution of neurons with longer neurites (Fig. 3*C*, df = 27, vehicle *p*-value = 0.0001, 1, 0.0001 and CPZ *p*-value = 0.09, 1, 1 for each bin respectively). To further assess the requirement for TRPV1, we tested DRG neurons from TRPV1 KO mice. Physiologic responses to capsaicin are completely absent in these mice (Caterina et al., 2000). DRG neurons from wild-type and TRPV1 KO mice were treated with capsaicin for 10 min and replated (as in Fig. 2*A*). Genetic loss of TRPV1 completely blocked capsaicin-induced outgrowth as assessed by mean neurite length (Fig. 3*D*, *E*, df = 9, WT *p*-value = 0.006, KO *p*-value = 1, WT vs. KO *p*-value = 0.009) as well as the population shift toward having longer neurites (Fig. 3*F*, df = 27, WT *p*-values = 0.0004, 1, 0.002, KO *p*-values = 1, 1, 1, WT versus KO *p*-values = 0.0004, 1, 0.004 for each bin, respectively). Importantly, TRPV1 KO DRG neurons grew normally in culture following dissection from the animal and were indistinguishable from wild-type neurons before drug treatment (data not shown) indicating that they have the ability to grow after injury. Therefore, the lack of outgrowth following capsaicin is not due to an inability of TRPV1 KO DRG neurons to grow. This demonstrates that axon outgrowth following capsaicin treatment is via TRPV1 activation.

### Capsaicin induces axon outgrowth in nociceptive neurons

Having demonstrated that TRPV1 is required for capsaicin-induced outgrowth, we next sought to determine if axon outgrowth was occurring in nociceptive neurons. We used neurofilament 200 (NF200) immunostaining to classify neurons as large-diameter, non-nociceptive neurons (NF200^+^; Frey et al., 2015). NF200-negative neurons displayed a 2.9-fold increase in mean neurite length (Fig. 4*A*,*B*, df = 23, *p* = 0.003). NF200-positive neurons did not show a significant increase in neurite length (Fig. 4*A*,*B*, df = 23, *p* = 1 compared to DMSO, *p* = 0.004 capsaicin NF200^+^ vs. NF200^–^). Similarly, NF200^–^ neurons showed a significant increase in the percentage of cells having longer neurites, while NF200^+^ neurons showed no change (Fig. 4*C*, df = 45, NF200^+^ *p*-values = 1, 1, 1, NF200^–^ *p*-values = 0.0001, 1, 0.0001, NF200^+^ vs. NF200^–^ *p*-values = 0.0001, 1, 0.0001 for each bin, respectively). These findings suggest that capsaicin induces a cell-autonomous pro-regenerative signal within nociceptors.

### TRPV1-induced axon outgrowth is not due to toxicity of poorly growing neurons

Nociceptive neurons do not regenerate as well as non-nociceptive neurons, and some reports demonstrate that they upregulate pro-death molecules in response to injury (Kalous and Keast, 2010; Hu et al., 2016; Berta et al., 2017). Furthermore, activation of TRPV1 can ablate TRPV1-expressing neurons and axon terminals (Mishra and Hoon, 2010; Wang et al., 2017). Therefore, we considered the possibility that capsaicin induces cell death via TRPV1 and eliminates poorly growing neurons from the population, thereby artificially skewing the distribution of neurons toward better axonal growth. To address this possibility, we assessed the health of DRG neurons after capsaicin treatment. Neurons were treated as in Fig 2*A*, but instead of replating neurons after 24 h, they were incubated with ethidium homodimer, a cell death marker. Viability was not affected by capsaicin treatment (DMSO = 88.1% and CAPS = 89.6% viable *n* = 3, df = 4, *p* = 0.8). To assess general health, neurons were also loaded with TMRM, a mitochondrial potential dye. Again, mitochondrial potential was not affected by capsaicin treatment (DMSO = 1 and CAPS = 1.01 normalized intensity, *n* = 3, df = 2, *p* = 0.8). We conclude that capsaicin is not negatively impacting the health of DRG neurons. This indicates that enhanced outgrowth following capsaicin treatment is not due to loss of poorly growing neurons.

### Capsaicin activates a pro-regenerative–like program

The pro-regenerative program works by upregulating pro-growth genes. SCG10 is an excellent regeneration marker because it is upregulated after injury, is specific to regenerating neurons, and is robustly elevated after injury *in vivo* and *in vitro* (Mason et al., 2002; Grenningloh et al., 2004; Shin et al., 2012b, 2014; Frey et al., 2015). We found that capsaicin upregulates SCG10 protein levels ∼1.8-fold (Fig. 5*A*,*B*, df = 4, *p* = 0.01), The distribution of SCG10 intensity revealed that only a small subset of neurons upregulated SCG10 (Fig. 5*C*). There is a decrease in the percentage of neurons with low-level expression (<1.25, df = 12, *p* = 0.005) and a subsequent increase in the percentage of neurons with robust SCG10 expression (>1.75, df = 12, *p* = 0.007). This is consistent with TRPV1 being activated in a subset of sensory neurons. Together, the induction of SCG10 and the promotion of axon outgrowth provides both molecular and functional evidence that capsaicin “reprograms” cultured dorsal root ganglion neurons into a regenerative-like state.

### Extracellular calcium is required for capsaicin-induced outgrowth

Having demonstrated that capsaicin promotes axon outgrowth, we next sought to define the mechanism by which TRPV1 activation leads to the induction of the pro-growth program. TRPV1 is permeable to both Na^+^ and Ca^2+^, and thus its activation depolarizes neurons thereby leading to an increase in intracellular calcium. Calcium is a known mediator of neuronal injury responses; injury triggers a calcium influx that is involved in membrane resealing, growth cone formation, and activation of the regenerative program (Bradke et al., 2012; Cho et al., 2013; Rishal and Fainzilber, 2014). It is unknown whether calcium influx via ligand-gated ion channel activation could mediate a regenerative response. Therefore, we tested whether extracellular calcium was required in this paradigm. Neurons were pretreated with EGTA for 10 min before capsaicin treatment. After capsaicin treatment, both drugs were washed out, and neurons were replated 24 h later and grown for 18 h (Fig. 6*A*). Chelating extracellular calcium with EGTA completely blocked both the increase in mean neurite length (Fig. 6*B*,*C*, df = 6, vehicle *p*-value = 0.004, EGTA *p*-value = 1, vehicle vs. EGTA *p*-value = 0.007) and the shift in population of more neurons having longer neurites (Fig. 6*D*, df = 18, vehicle *p*-values = 0.0001, 1, 0.0001, EGTA *p*-values = 1, 1, 1, vehicle vs. EGTA *p*-values = 0.002, 0.7, 0.0001 for each bin, respectively). These results indicate that there is a calcium-sensitive pathway downstream of TRPV1 activation that induces the pro-regenerative program. Importantly, EGTA did not affect baseline growth. This shows that EGTA is not blocking outgrowth via nonspecific damage to neurons.

### Capsaicin activates the PKA pathway to induce axon outgrowth

The cAMP/PKA pathway is stimulated by calcium influx and is well recognized for its ability to activate axon regeneration (Cai et al., 1999; Neumann et al., 2002; Cooper, 2015). To test whether capsaicin activates the PKA pathway, we assessed phosphorylation of the downstream, pro-regenerative transcription factor CREB. Neurons were pretreated with DMSO or the PKA inhibitor, H89, for 30 min, followed by capsaicin for 10 min. Inhibitors were present during the 10-min capsaicin treatment and for 20 min after capsaicin was removed (Fig. 7*A*). Then neurons were fixed and stained for phosphorylated CREB. Capsaicin induced a ∼2-fold increase in pCREB that was attenuated by PKA inhibition (Fig. 7*B*,*C*, df = 12, vehicle *p*-value = 0.003, PKAi *p*-value = 0.1). As with SCG10, pCREB was upregulated in only a subset of neurons (Fig. 7*D*, df = 27, vehicle *p*-values = 0.0001, 1, 0.0003, PKAi *p*-values = 0.1, 1, 1 for each bin, respectively).

Next, we tested the functional consequence of the PKA pathway in capsaicin-induced outgrowth. Neurons were pretreated with either DMSO or H89 for 30 min before the 10-min pulse of capsaicin. After capsaicin was removed, DMSO or H89 was added back (Fig. 8*A*). 24 h after capsaicin treatment, DMSO and H89 were removed, and neurons were replated and grown for 18 h in the absence of any drug. PKA reduced the 2.4-fold increase in outgrowth (Fig. 8*B*,*C*, df = 6, vehicle *p*-value = 0.0004, PKAi *p*-value = 0.5, vehicle vs. PKAi *p*-value = 0.009) and the shift in distribution of neurite lengths (Fig. 8*D*, df = 18, vehicle *p*-values = 0.002, 1, 0.0006, PKAi *p*-values = 0.09, 1, 0.1, vehicle vs. PKAi *p*-values = 0.2, 1, 0.02 for each bin, respectively). This indicates a role for the PKA pathway in capsaicin-induced axon growth. We propose that capsaicin activates TRPV1 to increase intracellular calcium, stimulating the PKA pathway to induce phosphorylation of CREB and initiation of the pro-regenerative program.

### Capsaicin induces regeneration signaling *in viv*o

Having defined the mechanism by which capsaicin induces axon outgrowth *in vitro*, we set out to determine if axon growth could be enhanced by activating the pro-regenerative program *in vivo*. Neurons preconditioned *in vivo* will exhibit robust growth in a short amount of time, for example 18 h, whereas uninjured neurons will not (Shin et al., 2012a). Indeed, we find that neurons that have been pre-injured display 200 ± 13 µm of growth in 18 h compared to only 32 ± 16 µm from uninjured neurons, a 6.3-fold increase (Fig. 9*A–C*, *n* = 3 per condition, df = 5, *p*-value = 0.0005). This robust increase in axon outgrowth is also observed as a decrease in the percentage of neurons with short axons and a subsequent increase in the percentage of neurons with long axons (Fig. 9*D*, df = 12, *p*-value < 0.0001, 1, <0.0001 for each bin, respectively). A common paradigm to determine if drugs induce a similar preconditioning-like response *in vivo* is to apply drug to the sciatic nerve then isolate DRGs a few days later and assess axon regeneration *in vitro* (Neumann et al., 2002; Valakh et al., 2015). We used this method to ask two questions: (1) does capsaicin induce a preconditioning like response *in vivo*? and (2) does capsaicin act locally on axons to induce regeneration? Since TRPV1 is expressed in the sciatic nerve, we applied 200 µm capsaicin directly to the sciatic nerve (Gold et al., 2003; Xing et al., 2012). 2 d later, DRGs were collected and cultured for 18 h to determine if treatment mimicked preconditioning (Fig. 9*A*). *In vivo* local capsaicin improved mean neurite outgrowth ∼1.5-fold over vehicle (Fig. 9*E*,*F*, df = 12, *p*-value = 0.004) and shifted a subset of neurons toward having longer neurites (Fig. 9*G*, df = 36, *p*-values = 0.008, 1, 0.02 for each bin, respectively). Capsaicin-induced axon outgrowth is not as robust as injury-induced outgrowth. This is expected, since capsaicin is inducing outgrowth in only a small subset of neurons, whereas injury will induce regeneration in most neurons. This finding confirms that the pro-regenerative pathway can be activated by capsaicin *in vivo*. We further tested whether capsaicin was sufficient to promote axon regeneration *in vivo.* We repeated the previous experiment but crushed the nerve instead of culturing the DRG 2 d after drug treatment. 24 h after crush, nerves were collected and analyzed. We did not observe improved regeneration in this paradigm (Fig. 9*H*). The measured regeneration index was 300 ± 71 and 390 ± 68 µm for DMSO and Capsaicin, respectively (df = 6, *p* = 0.4). These values are similar to what is observed from nerves that are not preconditioned (Shin et al., 2012a). This suggest that either capsaicin is not sufficient to activate enhanced regeneration in this fully *in vivo* paradigm or that this assay is not sensitive to changes in axonal growth in a small subpopulation of neurons.

## Discussion

After injury, long-distance regeneration requires a complex and coordinated pro-regenerative transcriptional program. However, this program has limitations. It is often too slow, and distal targets degenerate before reinnervation is achieved (Gordon et al., 2011). Furthermore, some cell types respond poorly or not at all. The preconditioning phenomenon illustrates that the pro-regenerative program can be augmented to improve regeneration. Activation of the program in DRG neurons by injury accelerates regeneration of the peripheral axon and allows for regeneration of the normally regeneration-resistant central axon (McQuarrie and Grafstein, 1973; McQuarrie et al., 1977; Richardson and Issa, 1984). We wished to identify pharmacological agents that mimic the preconditioning program in the absence of injury in hopes of promoting regeneration in regeneration-resistant neurons. We cultured neurons for several days to allow the initial injury signaling cascade, which is activated by dissection of the neurons from the animal, to turn off (Frey et al., 2015). This allowed us to test a drug’s ability to induce a regeneration response in the absence of ongoing injury signaling. This was an important element of our assay, because injury activates all pro-growth pathways and induces extremely robust regeneration. By replating neurons to assess axonal growth, we were also able to separate the “activation” phase from the “growth” phase and expose neurons to drugs only in the activation phase. This allowed us to select for drugs that induce a pro-regenerative program as opposed to drugs that might facilitate growth cone formation or cytoskeletal assembly. In this process, we discovered a method to stimulate known pro-regenerative pathways with a nociceptive ligand. Excitingly, nociceptive neurons are known to be poor at axon regeneration (Leclere et al., 2007; Kalous and Keast, 2010). The concept of targeting specific neuronal populations for regeneration via ligand activation may be generalizable.

Previous studies discovered that electrically stimulating neurons upregulated regeneration-associated genes and improved neurite outgrowth during stimulation (Goldberg et al., 2002). More recently, this technique has been tested *in vivo* and shown to mimic the preconditioning response (Senger et al., 2018). We discovered that the TRPV1 agonist, capsaicin, mimicked the preconditioning paradigm and induced axon outgrowth. This is unlike previous studies, since we can transiently stimulate a single receptor to mimic preconditioning. Stimulation of TRPV1 induces calcium influx and subsequent activation of PKA and the pro-regenerative transcription factor CREB. Furthermore, we show that local application of capsaicin to the sciatic nerve is sufficient to mimic a conditioning lesion. This indicates that activation of TRPV1 along the nerve can stimulate this pathway *in vivo*. Interestingly, we found that not all methods of calcium stimulation induced axonal outgrowth. Indeed, ionomycin fails to induce regenerative signaling or enhance neurite outgrowth (data not shown and Valakh et al., 2015). This suggests that TRPV1 may stimulate a signaling complex that is activated by local calcium influx. Scaffolding proteins can organize large complexes of signaling proteins with ion channels. In this case, the adaptor protein AKAP150 is an interesting candidate, as it can anchor PKA and adenylate cyclase and interacts with ion channels (Dell’Acqua et al., 2006; Efendiev et al., 2010). AKAP150 is expressed in 80% of TRPV1-positive neurons and physically interacts with TRPV1 (Schnizler et al., 2008; Efendiev et al., 2013). Therefore, it is possible that a TRPV1/AC/PKA/AKAP150 complex exists, and calcium influx from TRPV1 activation could locally activate this signaling module. We hypothesize that TRPV1 activation triggers a local influx of calcium, either directly or indirectly, to stimulate adenylate cyclase. Local cAMP production then activates PKA to turn on a pro-regenerative program via pCREB-dependent transcription.

The desire to regenerate non-nociceptive neurons is obvious. In the absence of these neurons, patients lose the ability to control their body and interact with their environment. The need to regenerate nociceptive neurons is less obvious but could be beneficial for multiple reasons. First, appropriate pain has its place. After injury, pain elicits guarding behaviors so that the injury is protected and given time to heal. In cases where individuals are unable to sense pain, such as diabetic neuropathy, they must monitor themselves for injury. Second, nociceptive neurons play an important role in neuroinflammation and vasodilation after tissue injury that facilitates wound healing (Barker et al., 2006; Chiu et al., 2012). Therefore, restoring function of nociceptors after injury could facilitate wound healing and prevent additional injuries.

Compared to large myelinated neurons, nociceptive neurons do not regenerate well (Leclere et al., 2007; Kalous and Keast, 2010), likely because of higher expression of growth inhibitory molecules as well as an inability to upregulate growth-promoting molecules (Gardiner et al., 2005; Christie et al., 2010). This study suggests that it may be possible to target regeneration-resistant neurons that express TRPV1 to overcome their inability to regenerate. Moreover, a deeper understanding of this pathway may identify methods to promote axonal regrowth from other regeneration-resistant neurons. The CNS does not regenerate after injury, yet it is susceptible to many types of axon injury (trauma, multiple sclerosis, white matter strokes). Interestingly, TRPV1 is expressed in some brain regions (Cavanaugh et al., 2011). If the components of this regeneration pathway are intact within the CNS, then this pathway may be useful for promoting regeneration in regeneration-resistant CNS neurons.

Prior studies have demonstrated that ion channel inhibition can promote axon regeneration (Tedeschi et al., 2016). Our work highlights the potential of extracellular ion channel ligands to activate known pro-regenerative pathways. Indeed, other ligands may induce complementary regeneration pathways and/or stimulate regeneration of additional neuronal cell types. In theory, with the proper complement of agonists, it may be possible to achieve targeted regeneration of damaged neurons while leaving unaffected neurons undisturbed.

## Conclusion

We provide evidence that stimulating TRPV1 mimics preconditioning and induces a pro-regenerative program mediated by calcium influx and PKA activation. PKA subsequently leads to phosphorylation of the pro-growth transcription factor CREB and upregulation of regeneration-associated proteins such as SCG10. This is the first example of an ion channel agonist inducing a signaling cascade to initiate regeneration. Ion channels are attractive therapeutic targets and could promote cell type–specific regeneration.

## Synthesis

Reviewing Editor: Mike Fainzilber, Weizmann Institute of Science Decisions are customarily a result of the Reviewing Editor and the peer reviewers coming together and discussing their recommendations until a consensus is reached. When revisions are invited, a fact-based synthesis statement explaining their decision and outlining what is needed to prepare a revision will be listed below.

The reviewing editor and an expert consultant examined the revised manuscript and the response to the J Neurosci reviewers. The consensus was that additional work is required before the manuscript can undergo full review. Specifically:

1) The authors explain why they were not able to evaluate whether TRPV1-signaling induces regeneration in TRPV1-expressing neurons, due to technical issues. It should however be feasible to use established markers for the main subtypes of DRG sensory neurons (at minimum nociceptive versus proprioceptive) to determine if they differ in outgrowth in response to capsaicin. This would provide at least a partial response on this issue.

2) The in vivo regeneration experiment requested by the reviewers should be done before re-review at eNeuro.

3) Much of the data is shown as binned values, dividing the neurons into three length categories. This mode of data presentation does not provide a clear picture on the variability and length distributions in each neuronal population. Please show the data using a presentation method that allows the reader better judgement of data variability and distributions.

4) When resubmitting, please make sure that you are responding on all the above points, and also all the issues raised by the previous reviewers at J Neurosci.
